# Pregabalin in Childhood Epilepsy: A Clinical Trial Study

**Published:** 2014

**Authors:** Mohsen MOLLAMOHAMMADI, Seyed Hassan TONKABONI, Zahra PIRZADEH, Mostafa Vahedian

**Affiliations:** 1 Pediatric Neurology Department, Hazrat Fatemeh Masoumeh Hospital, Qom University of Medical Sciences, Qom, Iran; 2 Pediatric Neurologist Research Center,Mofid Children’s Hospital, Shahid Beheshti University of Medical Sciences, Tehran, Iran; 3 Pediatric Neurology Center of Excellence, Department of Pediatric Neurology, Mofid Children Hospital, Faculty of Medicine, Shahid Beheshti University of Medical Sciences, Tehran, Iran; 4 Department of Pediatric Neurology, Qazvin University of Medical Sciences, Qazvin, Iran.; 5 MSc in Epidemiology, Clinical Development Research Center, Qom University of Medical Sciences, Qom, Iran

**Keywords:** Refractory seizures, Pregabalin, Epilepsy

## Abstract

**Objective:**

The prevalence of active epilepsy is about 0.5–1%, and approximately 70% of patients are cured with first anti-epileptic drugs and the remaining patients need multiple drugs. Pregabalin as an add-on therapy has a postive effect on refractory seizures in adults. To the best of our knowledge, there is no research with this drug in childhood epilepsy. We use pregabalin in children with refractory seizures as an add-on therapy. The objective of this study is to evaluate the effects of pregabalin in the reduction of seizures for refractory epilepsy.

**Material & Methods:**

Forty patients with refractory seizures who were referred to Mofid Children’s Hospital and Hazrat Masoumeh Hospital were selected. A questionnaire based on patient record forms, demographic data (age, gender,…), type of seizure, clinical signs, EEG record, imaging report, drugs that had been used, drugs currently being used, and the number of seizures before and after Pregabalin treatment was completed. We checked the number of seizures after one and four months.

**Results:**

After one month, 26.8% of patients had more than a 50% reduction in seizures and 14.6% of these patients were seizure-free; 12.2% had a 25–50% reduction; and approximately 61% had less than a 25% reduction or no change in seizures. After the fourth month, 34.1% of patients had more than a 50% reduction in seizures and 24.4% of these patients were seizure-free. Additionally, 65.9% of patients had less than 50% reduction in seizures (9.8% between 25–50% and 56.1% less than 25% or without improvement).

**Conclusion:**

We recommend Pregabalin as an add-on therapy for refractory seizures (except for myoclonic seizures) for children.

## Introduction

The prevalence of epilepsy is approximately 0.5–1.0% and about 70% of patients will become seizure-free from first–line anti-epileptic drugs (1-4). Refractory seizures are associated with cognitive and behavioral problems and impaired psychosocial development (5-7). 

There are many effective drugs effective for epilepsy, some of which are the main drugs and some are adjunctive drugs for treatment.

Pregabalin is an adjunctive drug and an alpha-2-delta ligand with anxiolytic, analgesic, and antiepileptic activity.

Pregabalin binds to the alpha-2-delta protein, which is an auxiliary protein associated with voltage-gated calcium channels; thereby, decreasing calcium influx at nerve terminals and, subsequently, the release of excitatory neurotransmitter glutamate.

Pregabalin has no efficacy at GABAa and GABAb receptors (8,9). 

Pregabalin has a pharmacological effect similar to GABApentin, but with a 3- to 10-fold increased potency (10).

There are many studies indicating that pregabalin is highly effective as an add-on therapy in adult patients with partial seizures, with or without secondary generalization (11-14).

There is little research concerning the effect of Pregabalin on childhood intractable epilepsy (15).

Pregabalin has a few side effects of which dizziness and somnolence are the most common (16).

## Materials & Methods

In this interventional research, we prescribed Pregabalin as an add-on therapy to children with refractory seizures. We selected 40 patients with refractory seizures who were referred to Mofid Children’s Hospital and Hazrat Masomeh Hospital.

A questionnaire based on the patient record form, demographic data (age, gender, among others), type of seizure, clinical signs, electroencephalography (EEG) record, imaging report, drugs that had been used, drugs currently being used, and the numbers of seizures before and after pregabalin treatment were recorded. 

Pregabalin of 5–15 mg/kg/day were prescribed. 

Follow-up on these patients was done for 4 months. Patients were visited after one month, and numbers of seizures were recorded and the patients were divided into 100%, 75–100%, 50–75%, <50%, and without response groups.

This study was registered as an Iranian clinical trials with registration number IRCT2014012216320N1 code.

## Results

The mean age of the patients was 74.46 ± 4.39 months (range 11–168 months). The first seizure was in 19.5 ± 2.4 Months. The mean duration of seizure was 51 ± 35 months (range 1–138 months). Fifteen (36.6%) patients were female and 25 (63.4%) were male (Table 1). The types of seizure were as follows: 22% tonic clonic, 22% complex partial seizure (CPS), and 56% mixed type seizure.

**Table 1:** The mean and standard deviation of age, duration of seizure, and age of first seizure

**Variable**	**Mean**	**Standard deviation**	**Minimum**	**Maximum**
**Age (months)**	74.46	4.39	11	168
**Duration (months)**	51	35	1	138
**Age of first seizure (months)**	19.5	24.2	1	120

An EEG was done for all patients, and 17.1% of patients had a normal EEG and the remainder had abnormal EEGs. All of these patients had neuroimaging and 53.7% showed normal results. 

Eight patients had more seizures before treatment with Pregabalin (4 per day).

After one month, 26.8% had more than a 50% reduction in seizures and 14.6% of these patients were seizurefree; 12.2% had between 25–50% reduction; and about 61% had less than 25% or no change in seizures. 

After four months, 34.1% of patients had more than a 50% reduction in seizures and 24.4% were seizure-free. Additionally, 65.9% had less than a 50% reduction in their seizures (9.8% between 25–50% and 56.1% less than 25% or without improvement). 

The mean rate of seizures before intervention was 13.17%, but this rate was 9.97% and 9.39%, one month and four month after pregabalin, respectively. 

**Table 2:** Mean Rate of Seizures Before and After Intervention with Pregabalin

	**Mean **	**S.D**
Before intervention	13.17	5.59
One month after intervention	9.97	6.18
Four month after intervention	9.39	7.07

**Fig 1:**
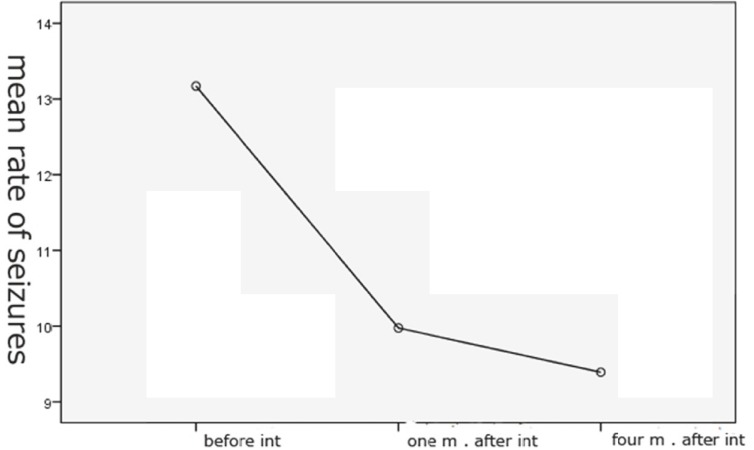
Effect of pregabalin on reduction of seizure

The results of ANOVA with repetitive measurements show a significant difference between the mean rate of seizures before and after intervention with Pregabalin (F=9.36, p=0.001) (Figure 1).

About 9.7% of patients had side-effects with headache was the most common (2.4%) and about 90.3% had no side-effects at all.

## Discussion

Pregabalin has been licensed as an antiepileptic drug for adjunctive therapy in adults with partial seizures with and without secondary generalization. 

Carreno et al. reported a 52% reduction of more than 50% in seizures with Pregabalin as an add-on therapy in adults with refractory partial seizures. Additionally, he reported a 60% adverse effect of which weight gain was the most frequent (14). In our research, all types of refractory seizures (partial and generalized) were experienced in childhood and we reduced seizures by about 34.1%.

Gil-Nagel et al. in a pooled data analysis of four randomized placebo-controlled trials confirmed the effect of Pregabalin in refractory partial seizures in adults, but more frequent side-effects were dizziness and somnolence, which is compatible with our findings (17). 

Jan et al. prescribed Pregabalin as add-on therapy in 19 children with refractory epilepsy and reported that 6% of children became seizure-free and 37% had more than a 50% reduction in seizures (15). The sample size of this research was small but the results were similar to our study. He prescribed Pregabalin 150–300 mg/day, but in our study, the drug was started with 2 mg/kg and then was increased to 10 mg/kg. 

There is no clear-cut evidence regarding the effective level and the toxic level of Pregabalin in children. 

A total of 34.1% of patients had more than a 50% reduction in seizure frequency after four months of treatment.

However, more trials with a greater sample size and long-term use of Pregabalin are needed to judge its long term effectiveness for different types of epilepsy. We recommend Pregabalin as add-on therapy in childhood refractory seizures, especially in patients with partial seizures and secondary generalized seizures. We suggest studying the pharmacokinetic of Pregabalin in childhood as a future study area.
